# Bidirectional ABO Mismatch Is Associated With Elevated Mortality in Hematopoietic Stem Cell Transplantation: Insights From a Single-Center Experience

**DOI:** 10.7759/cureus.54847

**Published:** 2024-02-24

**Authors:** Sidika Gülkan Özkan, Ali Kimiaei, Seyedehtina Safaei, Arif Ataberk Büyükyatıkçı, Meral Sönmezoğlu, Hasan Atilla Özkan

**Affiliations:** 1 Hematology, Bahçeşehir University, Istanbul, TUR; 2 Infectious Diseases, Yeditepe University Hospital, Istanbul, TUR

**Keywords:** major abo mismatch, bidirectional abo mismatch, pure red cell aplasia, allogenic stem cell transplant, hematopoietic stem cell transplantation

## Abstract

Background

Hematopoietic stem cell transplantation (HSCT) is a promising therapy for various disorders and provides new opportunities for patients. ABO incompatibility in allogeneic HSCT (allo-HSCT) remains a topic of debate because of its potential impact on clinical outcomes. This study aimed to analyze the survival outcomes of patients who underwent ABO-incompatible HSCT and evaluate the occurrence of pure red cell aplasia.

Methods

This retrospective study included 20 patients who underwent ABO-incompatible HSCT. Data on patient characteristics, transplant details, and follow-ups were collected. Conditioning regimens and graft-versus-host disease (GVHD) prophylaxis strategies were employed.

Results

Neutrophil and platelet engraftment durations did not differ significantly between major and bidirectional mismatches. Pure red cell aplasia occurred in 4 patients (20%) with major mismatches, all of whom responded well to bortezomib treatment. Patients with a bidirectional mismatch exhibited a 3.57-fold increase (hazard ratio [HR]: 0.28; p<0.05) in the risk of mortality compared to those in the major mismatch group.

Conclusion

The results indicate that ABO mismatch, whether bidirectional or major, does not significantly affect neutrophil and platelet engraftment duration, suggesting that ABO incompatibility may not be a major factor influencing hematological recovery in allo-HSCT. Interestingly, patients with bidirectional mismatch exhibited a significantly higher mortality rate than those with major mismatch. This finding suggests that a bidirectional ABO mismatch may have an unfavorable prognosis in terms of overall survival in allo-HSCT patients.

## Introduction

Hematopoietic stem cell transplantation (HSCT), a ray of hope and a potent therapy option, presents enormous promise for individuals suffering from a variety of disorders. HSCT has a wide range of applications, encompassing both malignant and benign diseases. Several factors can influence the outcome of allogeneic HSCT (allo-HSCT). These factors may be related to the donors and recipients. One of these factors is blood-type compatibility. Because of its potential impact on clinical outcomes, ABO incompatibility in allo-HSCT remains a hotly debated topic. ABO incompatibility occurs in a significant 23-40% of cases [1]. Notably, various studies have shed light on intriguing findings concerning ABO major-mismatched transplantation, revealing potential ramifications such as delayed red blood cell recovery, prolonged reliance on red blood cell transfusion, and an elevated risk of severe hemolytic complications and pure red cell aplasia (PRCA) [2-7]. However, it is important to note that conflicting reports exist, suggesting no significant correlation between ABO incompatibility and critical factors like overall survival (OS), transplant-related mortality (TRM), as well as neutrophil and platelet recovery [5]. Consequently, the definitive impact of the ABO mismatch on overall survival remains inconclusive, prompting further investigation and exploration in this intriguing area. This study aimed to analyze the survival outcomes of patients who underwent ABO-incompatible allo-HSCT at our institute. Our secondary goal was to explore the occurrence of PRCA and evaluate treatment strategies in this patient population.

## Materials and methods

Study design and data collection

This retrospective cohort study was conducted to evaluate the outcomes of 20 patients who underwent allo-HSCT at the adult bone marrow transplantation unit of our hospital between January 2017 and December 2021. Twenty of the 31 allo-HSCT cases were ABO-incompatible transplants, and blood group-incompatible transplants were compared. As there were no minor mismatched patient groups, the analysis was performed separately for major and bidirectional mismatched transplantations, considering various transplant characteristics to assess patient outcomes.

Patients, disease, and transplant characteristics

The study included 20 patients who underwent ABO-incompatible allo-HSCT with the following diagnoses: 11 with acute myeloid leukemia (AML), four with acute lymphoblastic leukemia (ALL), one with chronic lymphocytic leukemia (CLL), one with non-Hodgkin lymphoma (NHL), one with primary myelofibrosis (PMF), and two with myelodysplastic syndrome (MDS). Of the participants, 12 were female and eight were male (mean age: 54.5±14.5 years). The source of stem cells was peripheral blood in 19 cases (95%) and bone marrow in one patient (5%). The patients in our study were followed up for a median duration of 21.05 months, ranging from 3.9 to 57.6 months. During this period, regular follow-up assessments and evaluations were conducted to monitor progress, treatment response, and any potential complications or adverse events. The duration of follow-up varied among individuals, but the median duration of 21.05 months provides a representative measure of the observation period for the patients included in our study. Plasmapheresis was performed in one patient with a major ABO mismatch, while erythrocyte depletion was performed in another patient for hemolysis prophylaxis if the antibody levels were higher than 1/64 titer. Frozen products were used in 10 (59%) patients. ABO-incompatible terms were used based on the transfusion clinical guidelines. These guidelines culminate in a comprehensive review of authoritative sources, ensuring that they are based on the most up-to-date and reliable information available [8-10].

Conditioning regimens

At our center, the conditioning regimens used in allo-HSCT vary according to the specific protocols and patient characteristics. A reduced-intensity conditioning (RIC) approach was employed, utilizing various regimens tailored to individual requirements. These regimens included Fludarabine-Busulfan-Anti-thymocyte globulin (Flu-BU2-ATG), Flu-BU3, Thiotepa-Busulfan-Fludarabine (TBF), Cyclophosphamide-Fludarabine-Total Body Irradiation (Cy/Flu/TBI), Thiotepa-Cyclophosphamide-Fludarabine-Total Body Irradiation (Tio/Cy/Flu/TBI), Cyclophosphamide-Total Body Irradiation-Anti-thymocyte globulin (Cy/TBI/ATG), and Fludarabine-Melphalan (Flu/Mel), and one patient's regimen remains unknown.

Graft-versus-host disease (GVHD) prophylaxis

Our approach utilizes various GVHD prophylaxis strategies. Post-transplant cyclophosphamide was administered to 18 (90%) patients, anti-thymocyte globulin (ATG) was administered to seven (35%) patients, cyclosporine was administered to all patients, and mycophenolate mofetil (MMF) was used for 14 (70%) patients as part of GVHD prophylaxis. These combined approaches were carefully implemented to minimize the occurrence and severity of GVHD.

Statistical analysis

Statistical analyses were conducted using SPSS (version 24.0; IBM Corp., Armonk, USA). Descriptive statistics were used to summarize the study population's characteristics, presenting demographic and clinical features as percentages, means (±SD), or medians based on data distribution. Categorical variables were analyzed using frequencies and percentages. Comparative techniques, such as independent t-tests or Mann-Whitney U tests, were used to compare continuous variables between groups. Kruskal-Wallis, or one-way analysis of variance with Bonferroni correction, was used for analyses involving multiple groups. Correlation analyses were performed using Pearson’s or Spearman’s correlation tests. Associations between complications and other parameters were examined using chi-square or Fisher's exact tests. Survival analysis, using the Kaplan-Meier method, assessed longitudinal outcomes, with the log-rank test comparing survival curves between the study groups. Two-tailed p-values are reported, with a significance threshold set at 0.05.

## Results

Nine patients (45%) underwent allo-HSCT from haploidentical-related donors, including two with bidirectional ABO incompatibility and seven with major ABO incompatibility. Additionally, five patients (25%) received HSCT from 9/10 unrelated donors, comprising one with bidirectional ABO incompatibility and four with major ABO incompatibility. Three patients (15%) underwent HSCT from matched unrelated donors, two of whom had bidirectional ABO incompatibility, and one had major ABO incompatibility. Furthermore, two patients (10%) received HSCT from matched-related donors, with one exhibiting bidirectional ABO incompatibility and one demonstrating major ABO incompatibility. Lastly, one patient (5%) underwent HSCT from a 9/10 related donor and had bidirectional ABO incompatibility. Overall, there were seven transplants (35%) with bidirectional incompatibility and 13 transplants (65%) with major incompatibility.

During the follow-up period, which ranged from 3.9 to 57.6 months, with a median duration of 21.05 months, we investigated post-allo-HSCT blood group conversion. Among the patients with major ABO mismatches who underwent testing (n = 13), seven patients experienced a conversion of their red blood cell (RBC) phenotype to the donor's ABO phenotype, three patients retained their phenotype, two patients, unfortunately, passed away, and one patient's status could not be assessed due to dropout. Among the patients with bidirectional mismatches who underwent testing (n = 7), three patients underwent conversion of their RBC phenotype, one patient retained their phenotype, and three patients died. Patient demographics, transplant characteristics, and clinical outcomes are shown in Table 1.

**Table 1:** Patients' demographics, transplant characteristics, and clinical outcomes. AML: acute myeloid leukemia; ALL: acute lymphoblastic leukemia; CLL: chronic lymphocytic leukemia; NHL: non-Hodgkin lymphoma; PMF: primary myelofibrosis; MDS: myelodysplastic syndromes; 9/10 UD: 9/10 matched-unrelated donor; MUD: matched-unrelated donor; MRD: matched-related donor; 9/10 RD: 9/10 related donor; Flu: fludarabine; BU: busulfan; ATG: Anti-thymocyte globulin TBF: Thiotepa-Busulfan-Fludarabine; Tio: Thiotepa; Cy: Cyclophosphamide; TBI: Total body irradiation; Flu: Fludarabine; Mel: Melphalan; PT-Cy: post-transplant Cyclophosphamide; CsA: Cyclosporine A; MMF: Mycophenolate Mofetil. A p-value of less than 0.05 was considered statistically significant.

	All population, n=20	Compatibility		P-value	Survival		Pure red cell aplasia	
		Bidirectional incompatible	Major incompatible		Alive, n=10	Dead, n=10	No, n=15	Yes, n=4
Diagnosis, n (%)								
AML	11 (55)	3 (42.9)	8 (61.5)	0.506	6 (60)	5 (50)	8 (53.3)	3 (75)
ALL	4 (20)	1 (14.3)	3 (23.1)		2 (20)	2 (20)	3 (20)	0 (0)
CLL	1 (5)	1 (14.3)	0 (0)		1 (10)	0 (0)	1 (6.7)	0 (0)
NHL	1 (5)	0 (0)	1 (7.7)		0 (0)	1 (10)	1 (6.7)	0 (0)
PMF	1 (5)	1 (14.3)	0 (0)		0 (0)	1 (10)	1 (6.7)	0 (0)
MDS	2 (10)	1 (14.3)	1 (7.7)		1 (10)	1 (10)	1 (6.7)	1 (25)
Transplantation type, n (%)								
Haploidentical	9 (45)	2 (28.6)	7 (53.8)	0.313	3 (30)	6 (60)	7 (46.7)	1 (25)
9/10 UD	5 (25)	1 (14.3)	4 (30.8)		4 (40)	1 (10)	2 (13.3)	3 (75)
MUD	3 (15)	2 (28.6)	1 (7.7)		2 (20)	1 (10)	3 (20)	0 (0)
MRD	2 (10)	1 (14.3)	1 (7.7)		1 (10)	1 (10)	2 (13.3)	0 (0)
9/10 RD	1 (5)	1 (14.3)	0 (0)		0 (0)	1 (10)	1 (6.7)	0 (0)
Donor gender, n (%)								
Female	4 (20)	0 (0)	4 (30.8)	0.292	3 (30)	1 (10)	1 (6.7)	2 (50)
Male	16 (80)	7 (100)	9 (69.2)		7 (70)	9 (90)	14 (93.3)	2 (50)
Patient blood type, n (%)								
A+	4 (20)	3 (42.90)	1 (7.70)	<0.001	1 (10)	3 (30)	4 (26.70)	0 (0)
B+	4 (20)	4 (57.10)	0 (0)		1 (10)	3 (30)	4 (26.70)	0 (0)
B-	2 (10)	0 (0)	2 (15.40)		1 (10)	1 (10)	1 (6.70)	1 (25)
O+	10 (50)	0 (0)	10 (76.90)		7 (70)	3(30)	6 (40)	3 (75)
Donor blood type, n (%)								
A+	12 (60)	4 (57.10)	8 (61.50)	0.749	7 (70)	5 (50)	9 (60)	2 (50)
B+	3 (15)	2 (28.60)	1 (7.70)		1 (10)	2 (20)	2 (13.30)	1 (25)
A-	2 (10)	1 (14.30)	1 (7.70)		0 (0)	2 (20)	2 (13.30)	0 (0)
AB+	2 (10)	0 (0)	2 (15.40)		2 (20)	0 (0)	1 (6.70)	1 (25)
AB-	1 (5)	0 (0)	1 (7.70)		0 (0)	1 (10)	1 (6.70)	0 (0)
Compatibility, n (%)								
Bidirectional	7 (35)	7 (100)	0 (0)	-	1 (10)	6 (60)	7 (46.7)	0 (0)
Major	13 (65)	0 (0)	13 (100)		9 (90)	4 (40)	8 (53.3)	4 (100)
Regimen, n (%)								
Flu/BU2/ATG	5 (25)	1 (14.3)	4 (30.8)	0.390	2 (20)	3 (30)	3 (20)	2 (50)
Flu/BU3	2 (10)	0 (0)	2 (15.4)		2 (20)	0 (0)	1 (6.7)	1 (25)
TBF	3 (15)	1 (14.3)	2 (15.4)		1 (10)	2 (20)	2 (13.3)	1 (25)
Tio/Cy/Flu/TBI	3 (15)	2 (28.6)	1 (7.7)		1 (10)	2 (20)	3 (20)	0 (0)
Flu	1 (5)	1 (14.3)	0 (0)		0 (0)	1 (10)	1 (6.7)	0 (0)
Cy/TBI/ATG	2 (10)	1 (14.3)	1 (7.7)		1 (10)	1 (10)	2 (13.3)	0 (0)
Flu/Mel	1 (5)	1 (14.3)	0 (0)		1 (10)	0 (0)	1 (6.7)	0 (0)
NA	3 (15)	0 (0)	3 (23.1)		2 (20)	1 (10)	2 (13.3)	0 (0)
Plasmapheresis, n (%)								
No	19 (95)	7 (100)	12 (92.3)	0.999	9 (90)	10 (100)	14 (93.3)	4 (100)
Yes	1 (5)	0 (0)	1 (7.7)		1 (10)	0 (0)	1 (6.7)	0 (0)
Erythrocyte depletion, n (%)								
No	8 (40)	3 (42.9)	5 (38.5)	0.999	3 (30)	5 (50)	7 (46.7)	1 (25)
Yes	2 (10)	1 (14.3)	1 (7.7)		1 (10)	1 (10)	2 (13.3)	0 (0)
Frozen	10 (50)	3 (42.9)	7 (53.8)		6 (60)	4 (40)	6 (40)	3 (75)
Plasma depletion, n (%)								
No	9 (45)	3 (42.9)	6 (46.2)	0.569	4 (40)	5 (50)	8 (53.3)	1 (25)
Yes	1 (5)	1 (14.3)	0 (0)		0 (0)	1 (10)	1 (6.7)	0 (0)
Frozen	10 (50)	3 (42.9)	7 (53.8)		6 (60)	4 (40)	6 (40.0)	3 (75)
Stem cell source								
Peripheral blood	19 (95)	7 (100)	12 (92.3)	0.999	9 (90)	10 (100)	14 (93.3)	4 (100)
Bone marrow	1 (5)	0 (0)	1 (7.7)		1 (10)	0 (0)	1 (6.7)	0 (0)
PT-Cy, n (%)								
No	1 (5)	1 (14.3)	0 (0)		0 (0)	1 (10)	1 (6.7)	0 (0)
Yes	19 (95)	6 (85.7)	13 (100)		10 (100)	9 (90)	14 (93.3)	4 (100)
CsA, n (%)								
1	20 (100)	7 (100)	13 (100)	-	10 (100)	10 (100)	15 (100)	4 (100)
MMF, n(%)								
1	15 (75)	6 (85.7)	9 (69.2)	0.613	7 (70)	8 (80)	11 (73.3)	3 (75)
2	5 (25)	1 (14.3)	4 (30.8)		3 (30)	2 (20)	4 (26.7)	1 (25)
Neutrophil engraftment, n (%)								
Absent or lost	4 (20)	3 (42.9)	1 (7.7)	0.197	0 (0)	4 (40)	4 (26.7)	0 (0)
Present	16 (80)	4 (57.1)	12 (92.3)		10 (100)	6 (60)	11 (73.3)	4 (100)
Day	15 (10-23	12.5 (10-14)	16 (13-23)		15.5 (13-21	14 (10-23	14 (10-23	16.5 (14-21
Platelet engraftment, n (%)								
Absent or lost	6 (30)	3 (42.9)	3 (23.1)	0.682	1 (10)	5 (50)	5 (33.3)	0 (0)
Present	14 (70)	4 (57.1)	10 (76.9)		9 (90)	5 (50)	10 (66.7)	4 (100)
Day	14.5 (11-36	13 (11-16)	15 (11-36)		15 (11-36	13 (11-35	13 (11-35	17.5 (14-36
Survival, n (%)								
Alive	10 (50)	1 (14.3)	9 (69.2)	0.022	10 (100)	0 (0)	5 (33.3)	4 (100)
Dead	10 (50)	6 (85.7)	4 (30.8)		0 (0)	10 (100)	10 (66.7)	0 (0)
Blood group change, n (%)								
Yes	10 (50)	3 (42.9)	7 (53.8)	0.999	6 (60)	4 (40)	8 (53.3)	2 (50)
No	4 (20)	1 (14.3)	3 (23.1)		3 (30)	1 (10)	2 (13.3)	2 (50)
Dropout	1 (5)	0 (0)	1 (7.7)		1 (10)	0 (0)	0 (0)	0 (0)
Lost	5 (25)	3 (42.9)	2 (15.4)		0 (0)	5 (50)	5 (33.3)	0 (0)
Aplasia, n(%)								
No	15 (75)	7 (100)	8 (61.5)	0.256	5 (50)	10 (100)	15 (100)	0 (0)
Yes	4 (20)	0 (0)	4 (30.8)		4 (40)	0 (0)	0 (0)	4 (100)
Dropout	1 (5)	0 (0)	1 (7.7)		1 (10)	0 (0)	0 (0)	0 (0)

During the follow-up period, a total of ten patients, six with bidirectional mismatches and four with major mismatches, unfortunately, died. Causes of death in the major mismatch group included disease recurrence in three patients and an unknown cause of death in one patient. In the bidirectional mismatch group, there were three cases of sepsis, two instances of disease recurrence, and one unknown cause of death. The median number of days of neutrophil engraftment was found to be 12.5 days (range: 10-14 days) in patients with bidirectional mismatch and 16 days (range: 13-23 days) in those with major mismatch (p = 0.197). Furthermore, the median duration of platelet engraftment was 13 days (range, 11-16 days) in patients with bidirectional mismatch and 15 days (range, 11-36 days) in patients with major mismatch (p = 0.682). This indicated that there was no significant difference in the engraftment duration of platelets and neutrophils between major and bidirectional mismatched transplantations.

The incidence of PRCA was 20% (n = 4) and all patients exhibited major blood group incompatibility. A 100% response rate to bortezomib treatment has been observed in patients with PRCA, with a regimen consisting of administering 1.3 mg/m2 subcutaneously once per week for four weeks.

**Figure 1:**
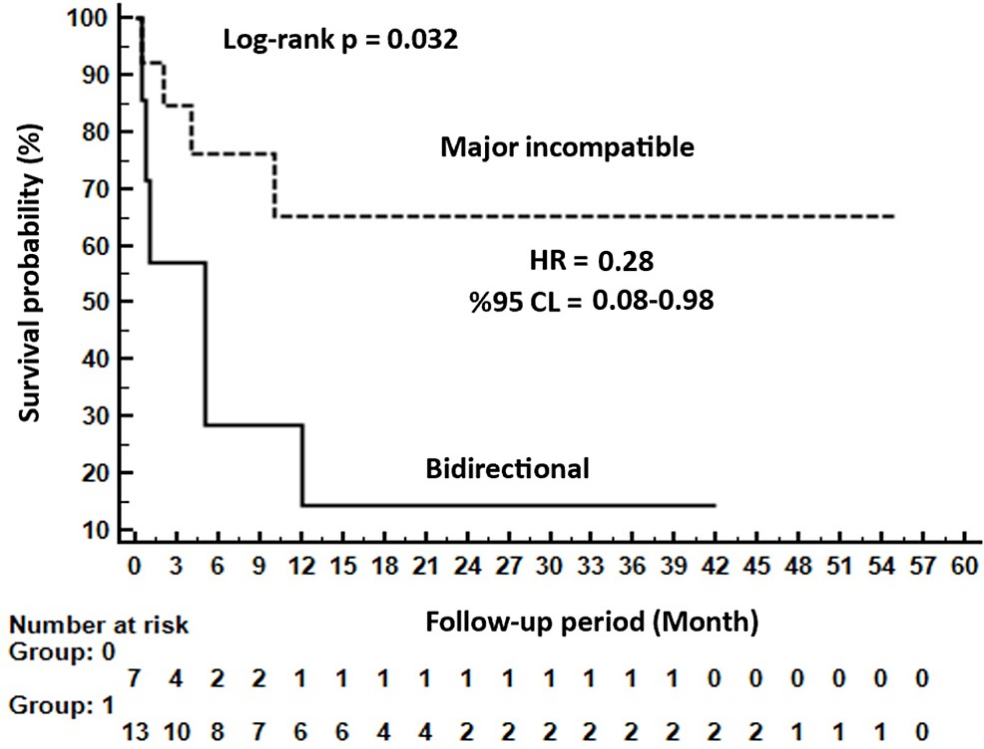
Kaplan-Meier survival analysis between major and bidirectional groups. HR: Hazard ratio; CL: Confidence intervals.

When comparing major mismatches with bidirectional incompatibility, the O+ and B− blood groups were more prevalent in major mismatches, and the mortality rate was significantly lower (85.7% vs. 30.8%; p = 0.022). Furthermore, patients with bidirectional incompatibility exhibited a 3.57-fold increase (HR: 0.28; p<0.05) in the risk of mortality compared with those in the major mismatch group. The Kaplan-Meier survival analysis between the major and bidirectional groups is shown in Figure 1.

## Discussion

This study aimed to analyze the survival outcomes of patients who underwent ABO-incompatible allo-HSCT and to explore the occurrence of PRCA and its treatment strategies in this patient population. Interestingly, bidirectional mismatch patients had a significantly higher mortality rate than those with major mismatches, indicating an increased risk of mortality in the former group. In addition, we found that neutrophil and platelet engraftment durations did not differ significantly between bidirectional and major mismatched transplantations. PRCA occurred in four patients (20%) with major mismatches, all of whom responded well to bortezomib treatment. These findings contribute to the understanding of ABO incompatibility in allo-HSCT and underscore its potential implications for patient outcomes and treatment strategies.

There are conflicting results regarding the effect of ABO compatibility on transplantation outcomes. According to a study that analyzed the impact of ABO mismatches on survival outcomes in various subgroups, including major, minor, and bidirectional mismatches, a decrease in survival was only evident in cases of minor ABO mismatches, whereas the overall survival rates of the major and minor mismatch groups were found to be inferior to those of the ABO-matched group [11]. In a comprehensive investigation involving a substantial cohort of over 3000 transplantation patients, a comparison between ABO-identical bone marrow transplantation (BMT) and ABO-incompatible BMT revealed no significant differences in survival outcomes [5]. Furthermore, Mielcarek et al. conducted a study involving 338 patients with ABO-incompatible HSCT and found no significant differences in either survival or GVHD between the ABO-incompatible groups [12]. One study suggests that ABO matching is not considered a critical factor for donor selection in various transplantation scenarios, including matched siblings, unrelated donors, and umbilical cord blood transplantation [13].

In contrast, other studies have shown that ABO incompatibility affects transplant outcomes. According to research conducted by Kanda et al., it was found that among related stem cell recipients, ABO matching had no significant influence on OS, while the minor and bidirectional mismatched groups among unrelated stem cell recipients exhibited lower OS with marginal significance, especially in patients with acute leukemia, patients who received transplants after 1998, and patients who underwent transplants at Asian centers [14]. Compared to ABO-identical HSCT, bidirectional ABO incompatibility was found to be significantly associated with an increased risk of mortality. However, no significant differences in mortality were observed between minor or major ABO-incompatible HSCT and ABO-identical SCT [3]. Moreover, previous studies have reported lower survival rates in patients undergoing bidirectional ABO-incompatible bone marrow transplantation (BMT) [3,4].

Topcuoglu et al. suggested that the increased mortality observed in bidirectional ABO-incompatible SCT may be partially attributed to the combined effects of major and minor ABO incompatibility, which could synergistically or additively enhance adverse outcomes. The major ABO barrier, which leads to cell damage and cytokine release shortly after transplantation, could potentially amplify the subsequent activation of the anti-host donor lymphocytes associated with the minor ABO barrier. Another possible explanation is that impaired survival is linked to an unidentified minor histoantigen, and bidirectional ABO incompatibility serves as a surrogate marker rather than a direct cause of this finding [3].

According to previous studies, PRCA can occur in approximately 7-8 percent of ABO-incompatible hematopoietic stem cell transplantations [15]. In a recent study involving 48 patients with PRCA, several risk factors for PRCA development were evaluated. These findings indicate that a major ABO mismatch is a significant risk factor [16]. These factors contribute to an increased likelihood of PRCA development in ABO-incompatible transplantation scenarios. In our study, we observed an incidence of PRCA in 20% of the cases (n = 4), with all patients exhibiting major blood group incompatibility. Importantly, all patients with PRCA showed a 100% response to bortezomib treatment. This highlights the potential efficacy of bortezomib in treating PRCA associated with major blood group incompatibility.

Despite these valuable findings, this study had several limitations. First, the sample size of 20 patients from a single center limits the generalizability of the results to a larger population. Additionally, the absence of a control group receiving ABO-compatible allo-HSCT makes it challenging to directly compare the outcomes and assess the specific impact of ABO incompatibility. Moreover, the underlying mechanisms and factors contributing to the observed outcomes, such as the influence of conditioning regimens or GVHD prophylaxis protocols, have not been extensively explored. Furthermore, the absence of data on GVHD and disease relapse limits the comprehensive assessment of the overall treatment efficacy and long-term survival outcomes. Furthermore, the study did not include patients with minor ABO incompatibility, which hinders a thorough examination of the outcomes associated with this specific type of mismatch. Finally, due to the retrospective nature of the study, anti-A, anti-B, and anti-AB titers were not available, which could have provided further insights into the dynamics of ABO incompatibility and its potential effects on transplant outcomes. Further studies with larger sample sizes, prospective designs, and comprehensive analyses are warranted to address these limitations and provide more conclusive evidence regarding the effects of ABO incompatibility in allo-HSCT.

## Conclusions

In conclusion, our study on ABO-incompatible HSCT provides valuable insights into survival outcomes, engraftment kinetics, blood group conversion, and the incidence of PRCA. Our findings revealed significant differences between patients with major and bidirectional mismatches. Interestingly, engraftment kinetics did not show significant differences between the two groups. The incidence of PRCA observed in our study aligns with that in the existing literature. Although the limited number of patients in our study precluded a comprehensive analysis of the risk factors for PRCA, our findings support the efficacy of bortezomib as a highly effective treatment for this condition. As allo-HSCT candidates may face challenges finding compatible donors for both ABO and HLA, ABO-incompatible allo-HSCT has become an unavoidable option. However, when possible, ABO-matched donors should be considered for allo-HSCT recipients. In cases where ABO matching is not feasible, we suggest that major incompatibility is preferable to a bidirectional mismatch. Furthermore, it is crucial to emphasize that patients with bidirectional ABO incompatibility should receive careful monitoring to optimize their outcomes.
